# An Extrinsic Optical Fiber Sensor Probe with Micrometer Size via a C-Shaped Waveguide with a Core of MIP

**DOI:** 10.3390/s25103250

**Published:** 2025-05-21

**Authors:** Chiara Marzano, Rosalba Pitruzzella, Francesco Arcadio, Filipa Sequeira, Luca Pasquale Renzullo, Alessandra Cutaia, Catarina Cardoso Novo, Ricardo Oliveira, Maria Pesavento, Luigi Zeni, Giancarla Alberti, Nunzio Cennamo, Rogerio Nunes Nogueira

**Affiliations:** 1 Department of Engineering, University of Campania Luigi Vanvitelli, Via Roma 29, 81031 Aversa, Italy; chiara.marzano@unicampania.it (C.M.); rosalba.pitruzzella@unicampania.it (R.P.); francesco.arcadio@unicampania.it (F.A.); lucapasquale.renzullo@unicampania.it (L.P.R.); luigi.zeni@unicampania.it (L.Z.); 2 Instituto de Telecomunicações, University of Aveiro, Campus Universitário de Santiago, 3810-193 Aveiro, Portugal; fsequeira@av.it.pt (F.S.); catarinacnovo@av.it.pt (C.C.N.); oliveiraricas@av.it.pt (R.O.); 3 Department of Chemistry, University of Pavia, Via Taramelli 12, 27100 Pavia, Italy; alessandra.cutaia01@universitadipavia.it (A.C.); maria.pesavento@optosensing.it (M.P.)

**Keywords:** reflection schemes, optical fiber sensors, molecularly imprinted polymers, optical–chemical sensors, 2-Furaldehyde (2-FAL)

## Abstract

Optical–chemical sensors based on optical fibers can be made in reflection or transmission schemes. In the reflection scheme, the sensing area is typically present at the end of the fiber, and the light source and the detector are placed on the same side of the fiber. This approach can be exploited to achieve chemical probes useful in several application fields where remote sensing is required. In this work, to obtain an extrinsic optical fiber chemical sensor in a reflection scheme, two optical fibers are used to monitor a chemically sensitive region achieved by a C-shaped waveguide with a molecularly imprinted polymer (MIP) as a core between the optical fibers. The proposed micrometer-sized probe is developed and tested as a proof of concept via a MIP for 2-Furaldehyde (2-FAL) detection of interest in food and industrial applications. The experimental results of the proposed sensing approach showed several advantages, such as a nanomolar detection limit and an ultra-wide concentration detection range due to different kinds of MIP recognition sites in the optical path between the fibers.

## 1. Introduction

A fiber-optic chemical sensor (FOCS) is a device designed to detect a sample’s specific chemicals or chemical properties, using light as the sensing medium [1]. This type of sensor takes advantage of light propagation through an optical fiber (OF) and the interactions between the light and the chemical sample under analysis [2].

Usually, the term FOCS is used when referring to chemical sensors that are intrinsic to optical fibers, i.e., in which the suitably modified OF interacts directly with the analyzed medium. In this case, FOCS systems typically include three basic components [2]. The first is an intrinsic active sensing unit that consists of an active optical fiber-performing molecule or ion detection through a transducer that generates an optical signal transmitted to the sensing system. The second component is a system for detecting optical signal parameters, such as intensity, frequency, or phase. These parameters carry information about the variables monitored by the sensor (e.g., refractive index, pH, and concentrations of chemicals or biochemicals) and convert them into processable electrical signals, such as current, voltage, or frequency. The third component is a control and evaluation system, which involves the use of computers and software tools for real-time data acquisition and analysis, ensuring intuitive operation and enabling interference-free measurement and online monitoring [2].

In recent years, another interrogation scheme based on sensor systems extrinsic to OFs, in which the sensor’s sensing region is not part of the OFs, has been emerging. In this case, the OFs are used to launch the light into the sensing and/or collecting region [3,4].

Intrinsic and extrinsic schemes based on OFs have significant differences that make them more suitable for specific applications [5]. Intrinsic schemes directly use an optical fiber as the sensing material and as a medium to carry the optical signal. In fact, the launching light never completely leaves the fiber except at the sensing end. Since the sensitive area is the OF itself, this scheme requires modifications to the OF to allow some light to escape, which involves several manufacturing processes [5]. On the other hand, extrinsic schemes use optical fibers only to transport light to an external zone, which is the actual sensing element. This approach allows for greater flexibility and the use of cheaper standard fibers instead of specialized fibers. In addition, having a separate sensing zone makes maintenance easier, as it is possible to interact only with the sensing zone without intervening on the whole system, and it also facilitates remote sensing [6,7].

Two interrogation schemes can be distinguished depending on the sensor’s optical configuration: reflection and transmission.

In the transmission scheme, light is transmitted through the sample from a transmitting OF to a receiving OF, outlining a longer optical path, allowing analysis of the properties of a sample along that optical path [8]. This feature makes it particularly suitable for applications that require a detailed analysis of the internal composition of the sample, such as spectroscopy and measurement of the concentration of substances in solutions. This approach is commonly used in benchtop instruments as it offers direct and controlled measurements, such as for point-of-care testing [9]. However, the transmission scheme has some limitations, for example, the need for precise alignment of the launching and collecting optical fibers. This requirement makes the probe unsuitable for measurements in places that are not easily accessible, such as well waters [10].

In the reflection scheme, the optical fiber is placed in front of a reflective surface or medium. The light emitted by the fiber is reflected from the surface and collected again by the same fiber or another coupled fiber (such as in this work). The amount of light reflected depends on various factors, such as the distance between the fibers, the type of reflective medium, or the properties of the surface itself. For example, it can be used to measure the position or distance of an object based on the amount of reflected light [11]. This type of sensor offers the advantage of easy installation and requires a single point of access. Typically, the receptor–analyte interaction occurs on the modified fiber tip in bio/chemical applications based on intrinsic optical fiber sensors [12,13].

The reflection scheme is preferred for remote or non-invasive measurements. A non-invasive approach has numerous advantages that make it particularly attractive in multiple fields. Non-invasiveness is particularly appreciated in the medical field, where it allows the diagnosis of diseases without resorting to invasive procedures [14,15,16], and in the environmental field, where samples can be analyzed without changing their characteristics. In addition, it allows repeated measurements over time without altering the initial conditions, enabling continuous monitoring of the state of the system. Finally, the reflection configuration facilitates installation and maintenance, as it requires access from only one side. The reflection scheme is an up-and-coming method for remote sensing, as the equipment and, therefore, the operator can be positioned at a distance from the sensitive area, reducing the risk of contamination [17].

In summary, the transmission scheme is a technique particularly useful in benchtop instrumentation. On the other hand, the reflection scheme makes these sensors suitable for remote applications or in harsh environments. The choice between the two schemes depends on the specific needs of the application and the characteristics of the sample to be analyzed [18,19].

In the bio/chemical sensing field, a FOCS is frequently coupled with a Molecularly Imprinted Polymer (MIP) as a recognition element [20,21,22,23,24]. MIPs are defined as synthetic receptors obtained by a template synthesis in which the analyte itself acts as a template. The molecular imprinting process occurs by preparing a polymeric mixture containing functional monomers, the target analyte (template), a cross-linker, and a radical initiator (if the polymerization is thermally performed) or an electrolyte (if the polymerization is electrically conducted). At the end of the synthesis, the template removal leaves recognition cavities in the polymer. These cavities have shapes and dimensions complementary to the analyte, making them suitable for selective rebinding. Unlike biological receptors, MIPs present several advantages, such as ease of preparation, storage stability, low cost, renewability, high mechanical strength, resistance to high temperature and pressure, and applicability in aggressive media [25,26]. Therefore, MIPs and their intrinsic polymer nature have attracted considerable attention in sensor production [27,28,29].

Despite the vast number of publications concerning MIP-based optical fiber sensors in transmission schemes [29,30,31], in the reflection scheme, only a few cases of FOCSs were exploited, despite the undoubted benefits [32].

This work focuses on proposing a simple extrinsic OF-based sensing principle that uses an inexpensive setup to ensure high performance in MIP–analyte interaction monitoring via a reflection scheme based on two OFs and the equipment located on the same side. Therefore, 2-Furaldehyde (2-FAL) [33] was used as a template in the MIP to develop a proof of principle. In short, 2-FAL is a furanic compound whose monitoring is important in various food and industrial applications [34,35,36].

In this scenario, the present work proposes a new optical–chemical sensor probe with micrometer size and simple implementation. In particular, two misaligned OFs are located in a C-shaped waveguide with a MIP-core to obtain an optically sensitive path between the optical fibers. In contrast to more complex techniques that exploit plasmons or other advanced optical phenomena on the fiber tip, our method is based on measuring the intensity of reflected light, offering a useful, versatile, and low-cost solution.

The proposed extrinsic OF sensing approach for reflection schemes was developed and tested, achieving promising results in terms of sensitivity, detection range, and selectivity.

## 2. Materials and Methods

### 2.1. Chemicals

2-Furaldehyde (2-FAL), methacrylic acid (MAA), divinylbenzene (DVB), 2,2′-azobisobutyronitrile (AIBN), Glyphosate (GLY), and 5-Hydroxymethylfurfural (5-HMF) were of analytical grade and obtained from Merck Life Science S.r.l., Milan, Italy. The MAA and the DVB were purified by solid phase extraction (SPE) with an aluminum oxide cartridge before their use.

### 2.2. MIP and NIP Preparation

The preparation of the pre-polymeric solution of MIP followed the procedure described in [37]. Briefly, MAA, the functional monomer, and DVB, the cross-linker, were mixed and sonicated to make a homogeneous solution. Next, the template, 2-FAL, was added in a quantity in such a way as to obtain a molar ratio of 2-FAL/MAA/DVB = 1/4/40. The mixture was then deaerated with a gentle nitrogen flow for ten minutes. Finally, AIBN (radical initiator, 20 mg/mL) was added to the solution. The non-imprinted polymer (NIP) was prepared similarly but without the template molecule.

### 2.3. Measuring Protocol

All experimental tests were performed according to the following protocol. Test solutions were deposited at a fixed volume of 500 μL per drop into a 3D-printed measurement cell housing the sensor. The standard deviation of the sensor response was calculated by evaluating the intensity value of reflected spectra at a specific wavelength recorded by adding and removing 20 times the blank solution, i.e., the solution without the analyte. This approach was repeated three times using similar sensor chips. The maximum measured standard deviation value was considered as the error bar within the dose–response curves. Subsequently, solutions with increasing concentrations of 2-FAL were incubated for a time sufficient to ensure the receptor–analyte interaction, equal to 10 min. After each incubation, washing steps with pure water were performed to eliminate non-specific interactions with the MIP, and the spectra for each sample were acquired in the same bulk solution, i.e., pure water. In this way, any intensity variation in the acquired spectra could be attributed only to the specific 2-FAL–MIP interaction.

To determine the sensor response for each concentration of 2-FAL, the intensity value of the blank solution was subtracted from the intensity value of the reflected spectrum at a fixed wavelength. In this way, for each 2-FAL solution, the sensor response (${\Delta I}_{fixed\;\lambda }$) was obtained. All spectra were visualized and recorded on a laptop through OceanView 2.0.16 software (by Ocean Insight, Orlando, FL, USA). Meanwhile, data analysis was performed using MATLAB (version R2024a, Mathworks, Natick, MA, USA) and OriginPro software (Origin Lab Corp., Northampton, MA, USA).

## 3. Sensor System Configuration

### 3.1. Production Steps of the Two-OFs-Based Sensor

**Figure 1:**
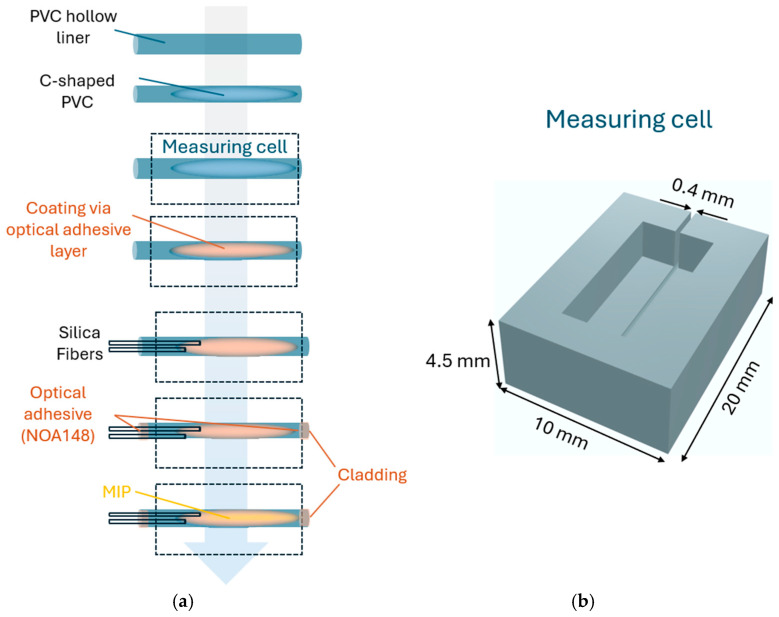
(**a**) Outline of the fabrication steps of the two-OFs-based sensor. (**b**) CAD drawing of the measuring cell with related measurements.

Figure 1a shows the steps required for the two-OFs-based sensor. First, a standard optical fiber (G657.A1) was removed from its PVC jacket, obtaining a hollow PVC liner with an inner diameter of approximately 300 µm, suitable for housing the sensing element. Through a manual cutting process, employing a sharp razor blade (Aeterna 191100, from Svenska Rakbladsfabriken, Grästorp, Sweden), approximately 5 mm of the outer region of the PVC jacket was removed. This allowed the hollow region of the PVC jacket to be exposed. The term C-shaped is relative to its cross-section. To guarantee that only the top region of the PVC jacket was removed and that the blade did not penetrate through the whole PVC jacket, an unclad silica fiber (125 µm diameter) was kept inside the hollow region of the PVC channel during the cutting process. After the first steps, in order to carry out and test the sensor, the PVC C-shaped channel was fixed inside a measurement cell designed via commercial CAD software (ver. Fusion 360, Autodesk, San Francisco, CA, USA) and printed using a resin-based 3D printer (Photon Mono X, Anycubic, Shenzhen, China). As shown in Figure 1b, the cell was 20 mm long, 10 mm wide, and 4.6 mm high. The measurement cell was created to facilitate experimental measurements and to contain the same volume (500 µL) of the test solutions during the incubation time (10 min).

As explained below, two silica OFs (GIF50C by Thorlabs, Newton, NJ, USA) were placed inside the hollow PVC C-shaped channel and coupled via a MIP-based sensitive waveguide. The silica OFs are multimodal graded-index fibers having a 50 µm core and a 125 µm cladding.

In the sensitive region, the offset between the fibers is necessary to collect only the reflection light via the MIP waveguide without collecting the direct light from the end of the input optical fiber.

In this way, the configuration called the “two-OFs-based sensor” was realized. After attaching the C-shaped PVC channel in the measurement cell, a coating of the PVC channel by NOA148 optical adhesive with a refractive index equal to 1.48 (supplied by Norland, Jamesburg, NJ, USA) was achieved via UV-curing before the optical fiber fixation step. This NOA148 layer was added to the PVC C-shaped channel because the MIP cannot adhere directly to the PVC. In addition, considering that the refractive index of the MIP (equal to approximately 1.61 RIU) is greater than that of the optical adhesive (equal to 1.48 RIU), the latter also acts as the cladding of the sensitive waveguide. Moreover, considering that the optical adhesive is hand-coated onto the PVC, its thickness is optically not relevant because it is sufficiently thick to act as cladding. After the coating step of the PVC channel, the optical fibers were placed into the channel and fixed by the same adhesive (NOA148). On the other side, the UV-cured optical adhesive (NOA148) was also used to cup the PVC channel at the end of the probe in order to ensure light reflection by acting as a cladding. Therefore, the UV-cured optical adhesive layers were exploited as the cladding of the optical MIP-based sensitive waveguide. Finally, the liquid MIP pre-polymeric mixture was dropped into the PVC channel, coated and cupped by the UV-cured optical adhesive, and thermally polymerized in an oven. In particular, the optical probe was placed in an oven at 55 °C overnight for thermal polymerization. After this, the template was removed by repeated washings with a pure ethanol solution.

### 3.2. Experimental Setup and Sensing Principle

**Figure 2:**
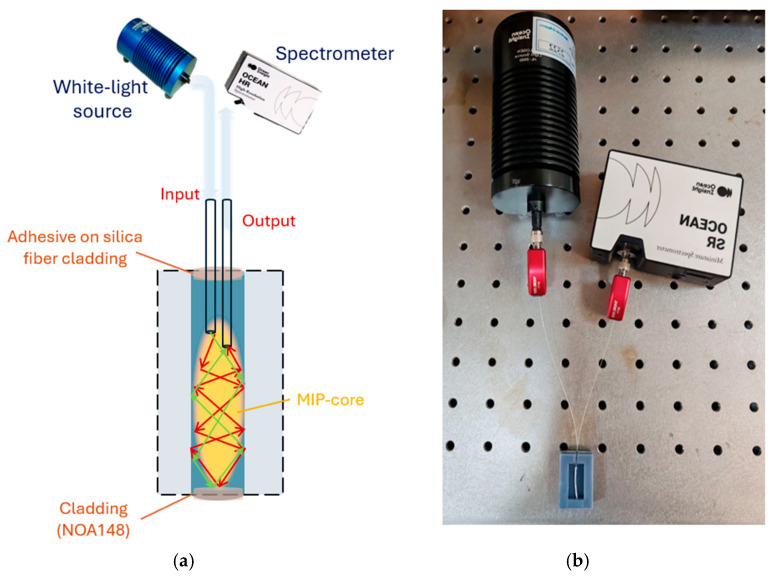
(**a**) Outline and (**b**) picture of the reflection-based experimental setup used for the two-OFs-based sensor.

The experimental setup consisted of simple elements: a halogen lamp (HL-2000-LL, Ocean Optics) and a spectrometer (SR-6VN500, Ocean Optics, Orlando, FL, USA) connected to a PC. The white light source had an emission range from 360 nm to 1700 nm, while the spectrometer had a detection range from 350 nm to 1023 nm. This two-OFs-based sensor was directly connected to the lamp on one side and the spectrometer on the other. The reflection spectra and data were displayed on the computer screen and saved by Ocean View software (version 2.0.16, Ocean Optics, Orlando, FL, USA). Analysis of the experimental data was performed using MATLAB and OriginPro software. Figure 2a shows an outline of the reflection-based experimental setup for the two-OFs-based sensor configuration under analysis, with the light optical path in the sensitive multimode waveguide. Figure 2b shows a picture of this setup.

As shown in the outline of Figure 2a, the offset between the fibers in the sensitive waveguide was used to collect the reflection light via the MIP-based waveguide (without collecting the direct light from the end of the input optical fiber). In the optical sensitive waveguide, the UV-cured optical adhesive (NOA148) layers were the cladding, and the MIP was the core of a multimode waveguide.

Regarding the sensing principle, when 2-FAL interacts with the MIP, 2-FAL changes the refractive index of the MIP, producing a change in the core of the waveguide and thus in the intensity of the propagated light, which is then reflected by the cladding (NOA148). The refractive index of the MIP increases when the analyte (2-FAL) binds the selective sites of the MIP [37], similarly to other MIP–analyte interactions [38]. When the refractive index of the MIP-core increases, the intensity of the propagated and reflected light also increases. The MIP-core sensing principle has also been exploited to develop other kinds of MIP-based optical fiber sensors via extrinsic and intrinsic schemes [39,40,41,42].

## 4. Results and Discussion

### 4.1. Dose–Response Curve of the Two-OFs-Based Sensor

**Figure 3:**
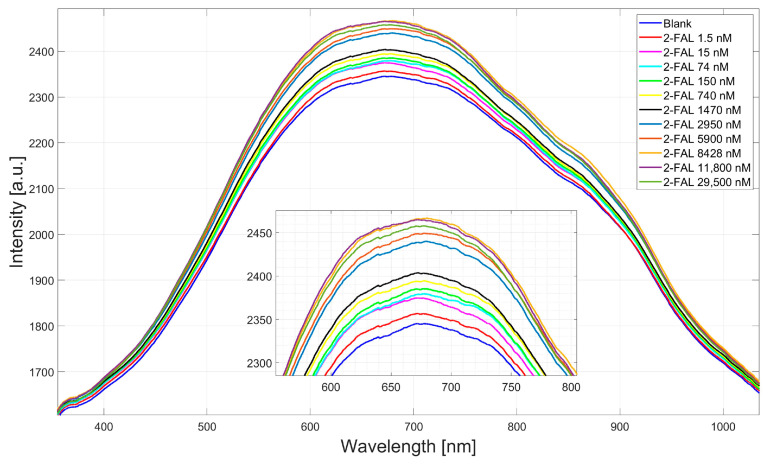
Reflected spectra intensity of the two-OFs-based sensor at different concentrations of 2-FAL solutions in water ranging from 1.5 nM to 29,500 nM.

The two-OFs-based sensor was tested with aqueous solutions at increasing concentrations of 2-FAL. Specifically, as shown in Figure 3, the range of tested concentrations was from 1.5 nM to 29,500 nM.

The intensity of reflected light increases with increasing analyte concentrations, as seen in Figure 3. This effect results from changes in the refractive index of the MIP caused by the specific interaction between 2-FAL and the polymer recognition sites.

**Figure 4:**
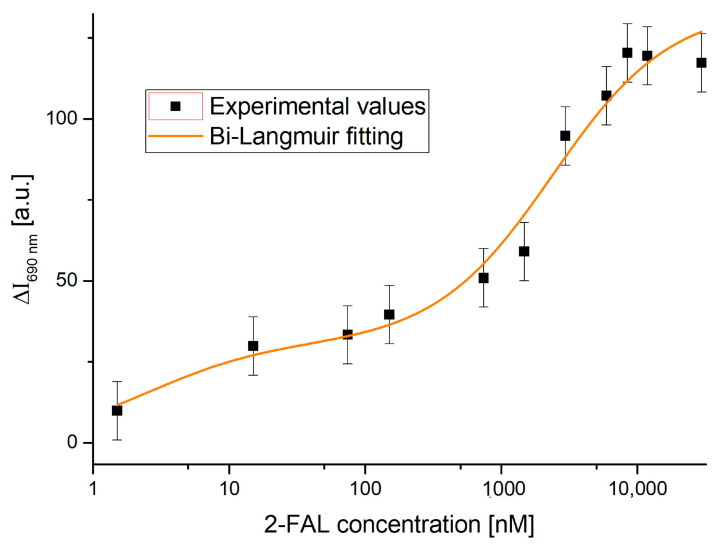
Dose–response curve of experimental values of the two-OFs-based sensor testing standard solutions with different 2-FAL concentrations. The experimental values together with the fit obtained by applying the Bi-Langmuir model (Equation (1)) and the error bar (maximum standard deviation of the sensor system) are shown in a semi-logarithmic scale.

The experimental values corresponding to the intensity variation (ΔI) caused by solutions with increasing concentrations of analyte versus blank at a fixed wavelength of 690 nm were obtained; since the effect is higher at this wavelength, the measurement should have a higher sensitivity. The results are reported in Figure 4, with the x-axis in a semi-logarithmic form, in order to be able to appreciate the whole considered concentration range. The Bi-Langmuir model was used to fit the experimental values, which assumes that two kinds of specific sites with different affinities for the template are present in this MIP [38]. The relationship is as follows (Equation (1)):

(1) $$\Delta I_{\left(690nm,c\right)}=\Delta I_{\left(690nm,cmax1\right)}\frac{c}{\left(K_{1}+c\right)}+\Delta I_{\left(690nm,cmax2\right)}\;\frac{c}{\left(K_{2}+c\right)}$$

where c corresponds to the concentration of the analyte and $K_{1}$ and $\Delta I_{\left(690nm,cmax1\right)}\;$ represent the affinity constant and the maximum intensity variation of the highest-affinity binding site (calculated at λ = 690 nm), respectively. Similarly, $K_{2}$ and $\Delta I_{\left(690nm,cmax2\right)}$ describe the parameters associated with lower-affinity binding sites at the same wavelength (690 nm).

The relationship in Figure 4 can be considered a dose–response curve for the determination of 2-FAL in aqueous solutions, since it represents the difference in intensity between solutions with increasing concentrations of the analyte and that of the blank at λ = 690 nm. The error bar corresponds to the maximum standard deviation of the sensor system (the worst case) relative to the blank solution, where there is no MIP–analyte interaction, obtained on three similar sensor chips (*n* = 20), equal to 9 a.u. Therefore, the experimental measurement error for each value is likely less than the reported error bar.

The fitting parameters related to the Bi-Langmuir model used to fit the experimental data in Figure 4 are reported in Table 1.

The $R^{2}$ value (equal to 0.97) shows that the Bi-Langmuir model fits the experimental data well in a wide concentration range.

It must be noted that the evaluation of $K_{1}$ is only indicative since the associated error is higher than 100%. On the contrary, the constant $K_{2}$, relative to the weaker sites, is significantly different from 0, with a high error of approximately 33%. From Table 1, the affinity constant of the MIP sites with the lowest affinity ($K_{aff2}$) can be calculated, as the $K_{aff2}$ is the inverse of $K_{2}$ and is equal to 0.42 [µM]^−1^ and it is similar to that obtained in the case of other 2-FAL sensors based on the same MIP combined with plastic OFs via different sensor configurations, such as those based on intensity variation [39,40] or exploiting plasmonic phenomena [37].

Considering only the first part of the standardization curve reported in Figure 4, in the concentration range from 1 nM to 100 nM, some analytical parameters of interest such as the sensitivity at low concentration ($S_{low\;c}$), the affinity constant ($K_{aff1}$), and the limit of detection (LOD) can be calculated. For this purpose, the Langmuir model is applied to the lower concentrations corresponding to the highest-affinity binding site in the Bi-Langmuir model. Equation (2) shows the Langmuir model employed [40]:

(2) $$\Delta I_{690nm,c}=I_{690nm,c}-I_{690nm,c_{0}}=\Delta I_{690nm,c_{max1}}\frac{c}{K_{1}+c}$$

In Equation (2), $K_{1}$ is the dissociation constant of the highest-affinity binding sites, i.e., those corresponding to the first part of Equation (1).

**Figure 5:**
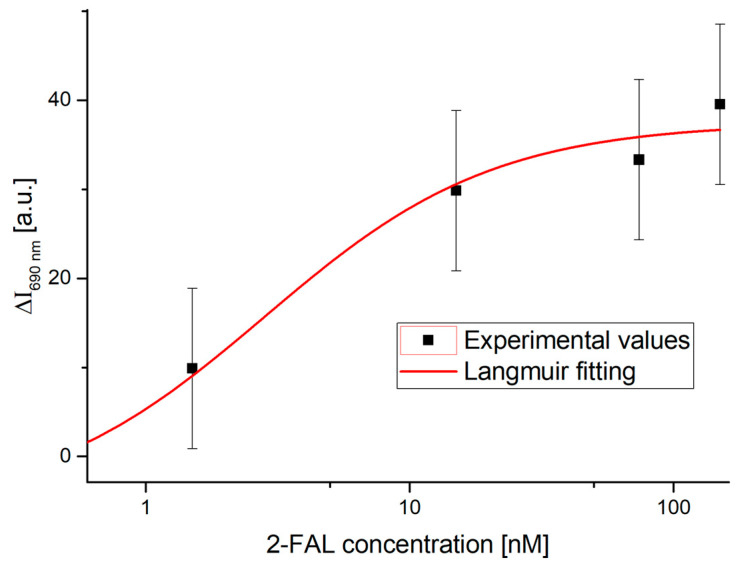
Dose–response curve relative to the highest-affinity binding sites achieved by solutions at a low concentration of 2-FAL. Experimental values with error bars are shown in a semi-logarithmic scale, and the red line represents the fitting of the data obtained by applying the Langmuir model (Equation (2)).

Figure 5 shows the dose–response curve fitted by this model.

The Langmuir fitting parameters, obtained using OriginPro software from the results plotted in Figure 5, are shown in Table 2.

In this case, $K_{1}$ is significantly different from 0, with a high error of 57%; therefore, some analytical parameters are also calculated for these sites at a higher affinity and reported in Table 3. More specifically, at low analyte concentrations (c<<K), Equation (2) becomes linear, and the slope, equal to ${\Delta I}_{690\;nm,\;cmax}/K$, is indicated as sensitivity at low concentration ($S_{low\;c}$), and is equal to 13.4 a.u./nM. This can be used to calculate the LOD being 1.5 nM [43]. The latter is calculated as the ratio between 3.3 times the standard error of the blank $\Delta I_{690\;nm,\;c0\;}$ (equal to 6 a.u.) and the $S_{low\;c}$. This is the minimum concentration that can be safely distinguished from 0, making it possible to state that 2-FAL is present in the considered sample. From Langmuir’s parameter $K_{1}$ given in Table 2, it is possible to derive the affinity constant $K_{aff1}$ of the high-affinity MIP sites monitored here ($K_{aff1}$ = 1/$K_{1}$), which is reported in Table 3, together with the other analytical parameters of interest. It should be noted that interaction sites with a high affinity in the MIP for 2-FAL were also detected by a different POF sensor previously proposed [42].

### 4.2. Selectivity Test of Two-OFs-Based Sensor

**Figure 6:**
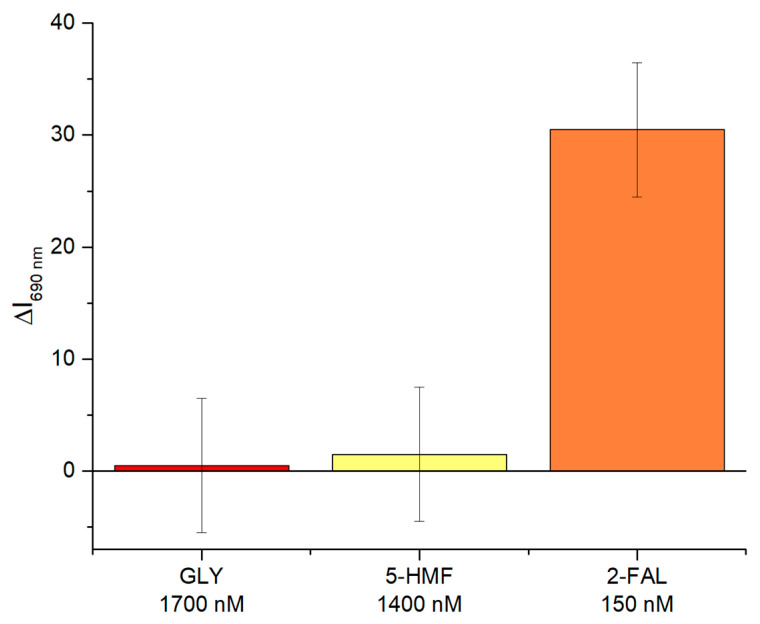
Selectivity test: obtained results by testing the proposed sensor with GLY (1700 nM), 5-HMF (1400 nM), and 2-FAL (150 nM).

To validate the selectivity of the two-OFs-based sensor, tests were conducted with substances other than the target analyte, which were Glyphosate (GLY) and 5-Hydroxymethylfurfural (5-HMF). These compounds were tested at significantly higher concentrations than 2-FAL, specifically 1700 nM for GLY and 1400 nM for 5-HMF, about an order of magnitude higher than the concentration of 2-FAL (150 nM) used in the test. Figure 6 shows the results obtained in the interference test.

As highlighted in Figure 5, adding these interfering substances did not cause significant changes in the sensor signal. In contrast, by adding the 2-FAL solution at a concentration of 150 nM, a significant variation was attained.

This result highlights the ability of the two-OFs-based sensor to specifically recognize 2-FAL, minimizing the interference of other chemical compounds and thus confirming the high selectivity of the developed detection system.

**Figure 7:**
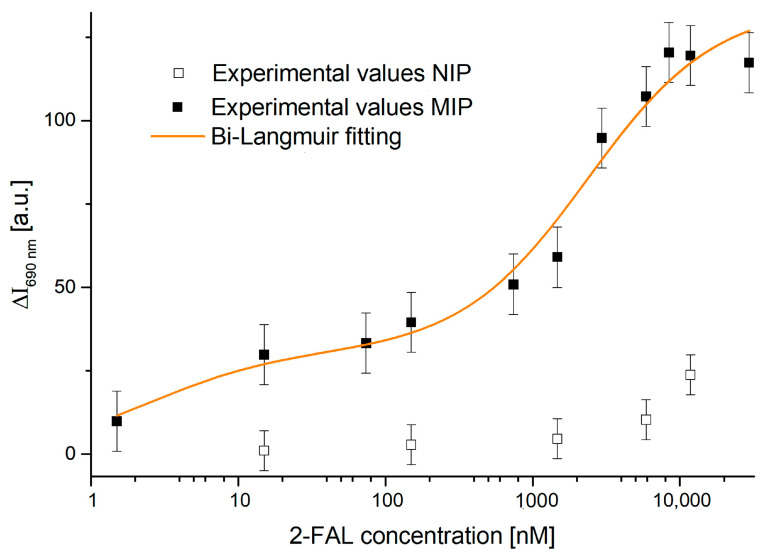
Detection of 2-FAL in water: comparison between the experimental values, with corresponding error bars, of MIP and NIP configurations of the two-OFs-based sensor. The solid line shows the fitting of the MIP sensor data using the Bi-Langmuir model (Equation (1)) on a semi-logarithmic scale.

In addition to the MIP sensor, a non-imprinted polymer (NIP) was prepared to confirm the selectivity of the proposed MIP sensor for 2-FAL. NIP was synthesized using the same procedure as MIP, described in Section 2.2, but without the presence of the 2-FAL template. This makes it possible to evaluate non-specific interactions between the polymer and analytes. Figure 7 shows the dose–response curve comparison of the MIP and NIP configurations of the two-OFs-based sensor in a semi-logarithmic scale.

As shown in Figure 7, the tested concentrations covered the full range analyzed with the MIP, making a full comparison possible. The NIP configurations of the two-OFs-based sensor did not show significant changes in intensity as the concentration of 2-FAL increased. The absence of a significant response with the NIP sensor confirms that the intensity changes observed with the MIP sensor are specifically due to the binding to 2-FAL and not to non-specific interactions with the polymer.

### 4.3. Discussion

To better position the proposed sensor within the current landscape, Table 4 reports on MIP-based 2-FAL optical fiber sensors, further highlighting the comparison with our state-of-the-art sensor.

When 2-FAL–MIP binding occurs, the MIP refractive index increases [37], producing a change in the waveguide’s core and increasing the intensity of the propagated light, similarly to [40], which then increases the reflected light.

A significant data point can be seen in Table 4 regarding the kinds of MIP sites. In particular, the calculated affinity constant associated with the MIP sites exhibiting the lowest affinity ($K_{aff2}$) for the proposed two-OFs-based sensor is very similar to that monitored in other sensor configurations based on the same MIP combined with plastic optical fibers (POFs) in transmission schemes [37,39,40]. This coherence in lower-affinity binding sites between different optical transduction methods underscores the robust and reproducible recognition capability of the synthesized MIP. The transmission-based sensor configuration [41] reported in Table 4 shows a significantly lower LOD due to MIP sites with a higher affinity, similar to the one achieved by the two-OFs-based sensor. However, the proposed sensor demonstrates a wider detection range (1.5 nM–8400 nM), exploiting two different kinds of MIP sites. Moreover, the obtained performance is especially significant considering the inherent advantages of the proposed reflection-scheme design, which offers a potentially simpler and more robust configuration by allocating both the spectrometer and the source on the same side.

## 5. Conclusions

This work developed a proof of concept of a micrometer-sized OF probe that operated according to a reflection interrogation scheme. The experimental results obtained with the developed MIP-based extrinsic OF sensor configuration demonstrated the presence of two kinds of receptor sites for 2-FAL with different affinities. Therefore, an ultra-wide 2-FAL detection range was obtained. The micrometer-sized probe (in total diameter) can be used to detect the substances of interest (2-FAL) in a reflection scheme by just connecting the probe’s OFs with a source and a detector.

The sensing approach used in this work, which exploits two OFs and MIPs, is notable for its simplicity and versatility compared with more complex OF-based techniques, such as those based on sensitive fiber tips. In particular, the presented extrinsic OF sensor is appropriate for use as a micrometer-sized probe in remote sensing systems, where small size and ease of implementation are critical for the detection of an analyte of interest at a nanomolar–micromolar concentration range. This novel approach offers the future possibility of measuring different target molecules simply by changing the MIP’s template, further expanding the range of applications of our device.

## Figures and Tables

**Table 1:** Fitting parameters related to the Bi-Langmuir model used to fit the experimental data.

$\Delta I_{\left(690nm,cmax1\right)}$ [a.u.]		$K_{1}$ [nM]		$\Delta I_{\left(690nm,cmax2\right)}$ [a.u.]		$K_{2}$ [nM]		Statistics	
Value	St. Error	Value	St. Error	Value	St. Error	Value	St. Error	Reduced Chi-Sqr	Adj. R-Square
31	6	2.5	2.8	104	8	2382	783	0.68	0.97

**Table 2:** Fitting parameters related to the Langmuir model used to fit the experimental data relative to the highest-affinity binding sites.

$\Delta I_{\left(690nm\right)}(c_{0})$ [a.u.]		$\Delta I_{\left(690nm\right)}(c_{\left(max,1\right)})$ [a.u.]		$K_{1}$ [nM]		**Statistics**	
Value	St. Error	Value	St. Error	Value	St. Error	Reduced Chi-Sqr	Adj. R-Square
−6	6	37	2	3	2	0.09	0.97

**Table 3:** Chemical parameters related to the binding sites with a higher affinity obtained from the Langmuir fitting values.

LOD [nM]	LOQ$\;=\;\frac{10\cdot \mathrm{LOD}}{3.3}$ [nM]	$S_{lowc}$ [a.u./nM]	$K_{aff1}=\frac{1}{K_{1}}$ [nM]^−1^
1.5	4.5	13.4	0.36

**Table 4:** Comparison analysis between 2-FAL sensors based on MIPs and optical fibers.

Sensor Configuration	Scheme	Range of Detection	LOD	Ref.
SPR–POF–MIP sensor	Transmission	0.120 μM–60.4 μM	120 nM	[37]
MIP-Splitter-based sensor	Transmission	0.52 μM–52 μM	520 nM	[39]
MIP-core waveguide	Transmission	0.01 μM–1.2 μM	10 nM	[40]
Micro OF-MIP-OF sensor	Transmission	1.5 nM–150 nM	1.5 nM	[41]
Two-OFs-based sensor	Reflection	1.5 nM–8.4 μM	1.5 nM	This work

## Data Availability

Data will be made available upon request.

## References

[1] Wang X., Wolfbeis O.S. (2019). Fiber-Optic Chemical Sensors and Biosensors (2015–2019). Anal. Chem..

[2] Pospíšilová M., Kuncová G., Trögl J. (2015). Fiber-Optic Chemical Sensors and Fiber-Optic Bio-Sensors. Sensors.

[3] Cennamo N., Zeni L., Pesavento M., Marchetti S., Marletta V., Baglio S., Graziani S., Pistorio A., Ando B. (2019). A Novel Sensing Methodology to Detect Furfural in Water, Exploiting MIPs, and Inkjet-Printed Optical Waveguides. IEEE Trans. Instrum. Meas..

[4] Arcadio F., Zeni L., Perri C., D’Agostino G., Chiaretti G., Porto G., Minardo A., Cennamo N. (2021). Bovine Serum Albumin Protein Detection by a Removable SPR Chip Combined with a Specific MIP Receptor. Chemosensors.

[5] Pendão C., Silva I. (2022). Optical Fiber Sensors and Sensing Networks: Overview of the Main Principles and Applications. Sensors.

[6] Matějec V., Barton I., Pospisilova M., Traplova L. (2019). Extrinsic Fiber-Optic Sensor for Detection of Saliva pH. Chem. Afr..

[7] Arcadio F., Zeni L., Minardo A., Eramo C., Di Ronza S., Perri C., D’Agostino G., Chiaretti G., Porto G., Cennamo N. (2021). A Nanoplasmonic-Based Biosensing Approach for Wide-Range and Highly Sensitive Detection of Chemicals. Nanomaterials.

[8] Euser T.G., Chen J.S.Y., Scharrer M., Russell P.S.J., Farrer N.J., Sadler P.J. (2008). Quantitative Broadband Chemical Sensing in Air-Suspended Solid-Core Fibers. J. Appl. Phys..

[9] Soares M.S., Vidal M., Santos N.F., Costa F.M., Marques C., Pereira S.O., Leitão C. (2021). Immunosensing Based on Optical Fiber Technology: Recent Advances. Biosensors.

[10] Ruiz-Córdova G.A., Vega-Chacón J., Sotomayor M.d.P.T., Tuesta J.C., Khan S., Picasso G. (2024). Development of an Optical Sensor Using a Molecularly Imprinted Polymer as a Selective Extracting Agent for the Direct Quantification of Tartrazine in Real Water Samples. Polymers.

[11] Leysen W., Gusarov A., Mégret P., Wuilpart M. (2019). Assessment of the Environmental Effects on the ITER FOCS Operating in Reflective Scheme with Faraday Mirror. Fusion Eng. Des..

[12] Kong L.-X., Zhang Y.-X., Zhang W.-G., Zhang Y.-S., Yan T.-Y., Geng P.-C., Wang B. (2019). Lab-on-Tip: Protruding-Shaped All-Fiber Plasmonic Microtip Probe toward in-Situ Chem-Bio Detection. Sens. Actuators B Chem..

[13] Li Y., Xin H., Zhang Y., Li B. (2021). Optical Fiber Technologies for Nanomanipulation and Biodetection: A Review. J. Light. Technol..

[14] Phillips J.P., Langford R.M., Chang S.H., Maney K., Kyriacou P.A., Jones D.P. (2010). Cerebral Arterial Oxygen Saturation Measurements Using a Fiber-Optic Pulse Oximeter. Neurocrit. Care.

[15] O’Hara J.A., Hou H., Demidenko E., Springett R.J., Khan N., Swartz H.M. (2005). Simultaneous Measurement of Rat Brain Cortex PtO2Using EPR Oximetry and a Fluorescence Fiber-Optic Sensor during Normoxia and Hyperoxia. Physiol. Meas..

[16] Liu L., Hao F., Morgan S.P., Correia R., Norris A., Korposh S. (2019). A Reflection-Mode Fibre-Optic Sensor for Breath Carbon Dioxide Measurement in Healthcare. Sens. Bio-Sens. Res..

[17] Goswami K., Klainer S.M., Dandge D.K., Thomas J.R. (2017). Fiber optic chemical sensors (FOCS): An answer to the need for small, specific monitors. Biosensor Technology.

[18] Silveira M., Frizera A., Leal-Junior A., Ribeiro D., Marques C., Blanc W., Díaz C.A. (2020). Transmission–Reflection Analysis in High Scattering Optical Fibers: A Comparison with Single-Mode Optical Fiber. Opt. Fiber Technol..

[19] Zhou F., Su H., Li X., Wen Z. (2018). Reflection and Transmission Spectra of Dynamic Optical Fiber Gratings. Opt. Quantum Electron..

[20] Haupt K., Mosbach K. (2000). Molecularly Imprinted Polymers and Their Use in Biomimetic. Sens. Chem. Rev..

[21] Kriz D., Ramstrom O., Mosbach K. (1997). Molecular Imprinting-New Possibilities for Sensor Technology. Anal. Chem..

[22] Haghdoust S., Arshad U., Mujahid A., Schranzhofer L., Lieberzeit P.A. (2021). Development of a MIP-Based QCM Sensor for Selective Detection of Penicillins in Aqueous Media. Chemosensors.

[23] Alizadeh T., Akhoundian M. (2010). A Novel Potentiometric Sensor for Promethazine Based on a Molecularly Imprinted Polymer (MIP): The Role of MIP Structure on the Sensor Performance. Electrochim. Acta.

[24] Tse Sum Bui B., Haupt K. (2010). Molecularly Imprinted Polymers: Synthetic Receptors in Bioanalysis. Anal. Bioanal. Chem..

[25] Kumar A., Mahato K. (2024). Recent Advancements in Bioreceptors and Materials for Biosensors. Biosensors in Precision Medicine.

[26] Owens P. (1999). Molecular Imprinting for Bio- and Pharmaceutical Analysis. TrAC Trends Anal. Chem..

[27] Alberti G., Zanoni C., Magnaghi L.R., Biesuz R. (2024). Molecularly Imprinted Polymers (MIPs). Sensory Polymers.

[28] Wu L., Li X., Miao H., Xu J., Pan G. (2021). State of the Art in Development of Molecularly Imprinted Biosensors. VIEW.

[29] Cennamo N., Pesavento M., Arcadio F., Marzano C., Zeni L. (2024). Advances in Plastic Optical Fiber Bio/Chemical Sensors to Realize Point-of-Care-Tests. TrAC Trends Anal. Chem..

[30] Xin X., Liu H., Zhong N., Zhao M., Zhong D., Chang H., Tang B., He Y., Peng C., He X. (2022). A Highly Sensitive Plastic Optic-Fiber with a Molecularly Imprinted Polymer Coating for Selective Detection of 4-Chlorophenol in Water. Sens. Actuators B Chem..

[31] Cennamo N., Pesavento M., Zeni L. (2021). A Review on Simple and Highly Sensitive Plastic Optical Fiber Probes for Bio-Chemical Sensing. Sens. Actuators B Chem..

[32] Wu N., Feng L., Tan Y., Hu J. (2009). An Optical Reflected Device Using a Molecularly Imprinted Polymer Film Sensor. Anal. Chim. Acta.

[33] Hoydonckx H.E., Van Rhijn W.M., Van Rhijn W., De Vos D.E., Jacobs P.A. (2007). Furfural and Derivatives. Ullmann’s Encyclopedia of Industrial Chemistry.

[34] Matharage S.Y., Liu Q., Wang Z., Walker D. Effect of Paper Type and Water Content in Paper on the Partitioning of 2-FAL between Liquid and Paper Insulations. Proceedings of the 2020 IEEE International Conference on High Voltage Engineering and Application (ICHVE).

[35] Nezami M.D.M., Wani S.A., Khan S.A., Khera N., Sohail S. (2018). An MIP-Based Novel Capacitive Sensor to Detect 2-FAL Concentration in Transformer Oil. IEEE Sens. J..

[36] Ansari M.T.I., Raghuwanshi S.K., Kumar S. (2023). Recent Advancement in Fiber-Optic-Based SPR Biosensor for Food Adulteration Detection—A Review. IEEE Trans. Nanobioscience.

[37] Pesavento M., Zeni L., De Maria L., Alberti G., Cennamo N. (2021). SPR-Optical Fiber-Molecularly Imprinted Polymer Sensor for the Detection of Furfural in Wine. Biosensors.

[38] Renzullo L.P., Tavoletta I., Alberti G., Zeni L., Pesavento M., Cennamo N. (2024). Plasmonic Optical Fiber Sensors and Molecularly Imprinted Polymers for Glyphosate Detection at an Ultra-Wide Range. Chemosensors.

[39] Tavoletta I., Arcadio F., Renzullo L.P., Oliva G., Del Prete D., Verolla D., Marzano C., Alberti G., Pesavento M., Zeni L. (2024). Splitter-Based Sensors Realized via POFs Coupled by a Micro-Trench Filled with a Molecularly Imprinted Polymer. Sensors.

[40] Arcadio F., Prete D.D., Zeni L., Pesavento M., Alberti G., Marletta V., Andò B., Cennamo N. (2024). Optical Waveguides Based on a Core of Molecularly Imprinted Polymers: An Efficient Approach for Chemical Sensing. IEEE Sens. J..

[41] Cennamo N., Arcadio F., Zeni L., Alberti G., Pesavento M. (2022). Optical-Chemical Sensors Based on Plasmonic Phenomena Modulated via Micro-Holes in Plastic Optical Fibers Filled by Molecularly Imprinted Polymers. Sens. Actuators B Chem..

[42] Pitruzzella R., Marzano C., Arcadio F., Sequeira F., Cutaia A., Cardoso Novo C., Scarpellini A., Alberti G. (2025). Silica Optical Fibers Connected via a Micro MIP-Core Waveguide to Build Optical-Chemical Sensors. Chemosensors.

[43] Thompson M., Ellison S.L.R., Wood R. (2002). Harmonized Guidelines for Single-Laboratory Validation of Methods of Analysis (IUPAC Technical Report). Pure Appl. Chem..

